# Dry Eye Disease and Refractive Corrections

**DOI:** 10.1155/2019/2058618

**Published:** 2019-12-06

**Authors:** David Madrid-Costa, James S. Wolffsohn, Javier Ruiz-Alcocer, Pablo De Gracia

**Affiliations:** ^1^Complutense University of Madrid, Madrid, Spain; ^2^Aston University, Birmingham, UK; ^3^Midwestern University, Chicago, IL, USA

Between the early 90s and 2004, more than 17 million patients received LASIK surgery worldwide. Nowadays, almost one million patients undergo corneal refractive surgery in the United States of America every year [1]. In addition, cataract surgery has become the most common surgical procedure [2, 3]. At the same time, the number of contact lens wearers is on the rise with a worldwide estimated number of around 120–140 million. Furthermore, the growing popularity of corneal refractive surgery, intraocular lens implantation, and contact lens use provokes that the number of patients that undergo several of these treatment modalities has also risen. This creates scenarios where tear film physiology is dramatically affected by one or several of the abovementioned procedures. In turn, this increases the risk of developing dry eye disease (DED). The prevalence of DED naturally increases with age, and the abovementioned treatments are often used to alleviate conditions in elderly patients such as cataracts or presbyopia.

Different types of refractive treatments can induce changes in tear film stability, an intensification of the ocular signs related to dry eye and symptoms, and an exacerbation of DED. The reverse pathway also occurs; an alteration on the ocular surface and tear film compromises the success of the abovementioned techniques for vision correction by altering the final visual quality and comfort of patients. The two-way interactions between several vision restoration techniques and DED have a worldwide impact that is on the rise.

DED and vision correction are hot topics on the field of vision science research and are key to public health organizations. This special issue aimed at focusing on novel approaches to treat and manage DED, advances in refractive corrections, and in the interrelations between both of these factors. Accordingly, this special issue gathered seven articles that addressed different aspects related to this hot topic.

In order to contextualize the current research efforts in the DED field and to know which are the most relevant topics, Sanchez-Tena et al. performed a citation network study and concluded that the definition and classification of DED followed by its treatment are the most researched area in this field. Regarding DED treatment approaches, Sakane et al. have presented the effects of a commercially available ophthalmic suspension (mucin secretagogue) on the quality of life of Japanese patients with DED.

It is very well known that DED is a multifactorial condition that can be exacerbated by systemic conditions. Wang et al. investigated the incidence, severity, and influencing factors of DED in systemic lupus erythematosus patients without secondary Sjögren's syndrome. Their conclusions stress the importance of monitoring the ocular surface of systemic lupus erythematosus patients and the early diagnosis of DED for improving the quality of life of these patients.

DED coexists sometimes with other ocular diseases aggravating its symptomatology and evolution. One clear example of these two-way interactions is found when keratoconus patients suffer greater symptoms of DED. Meibomian gland dysfunction (MGD), with a higher prevalence in keratoconus patients, plays a central role in DED and keratoconus. Meibomian glands heavily contribute to a healthy tear film, so when they are dysfunctional, the tear film is affected and eye rubbing normally increases. Very interestingly, eye rubbing is one of the mechanical etiological factors in keratoconic disease. Moreover, DED or tear film instability influences the success of the refractive correction of patients with keratoconus. Exploring these interactions, Mostafa et al. used noncontact meibography to examine the morphological changes in the meibomian glands of patients with keratoconus and its relationship with tear film parameters.

Blink rate affects tear film stability, and it can affect both DED symptomatology and the success of the refractive correction. This topic was addressed in two studies. García-Montero et al. reported the influence of different blink rate patterns on the tear film and on optical quality dynamics with different contact lens materials. Additionally, Itokawa et al. investigated the association between ocular surface temperature, tear film stability, and blink rate in patients after cataract surgery and concluded that blink rate may be a useful parameter for evaluating tear film stability in postcataract surgery patients.

In summary, this special issue identifies the research hot topics and reports information on novel treatments for DED, explores the association between DED and other ocular and systemic diseases, and explores the interactions between blink rate, ocular surface temperature, contact lens materials, tear film stability, cataract surgeries, and optical quality dynamics. Hence, we believe this special issue provides extremely useful knowledge intimately related to *Dry Eye Disease and Refractive Corrections.*
